# Predictors of non-suicidal self-injury in adolescents with depressive disorder: the role of alexithymia, childhood trauma, and body investment

**DOI:** 10.3389/fpsyg.2024.1336631

**Published:** 2024-04-04

**Authors:** Panpan Cao, Ran Peng, Qiuyu Yuan, Ruochen Zhou, Mengting Ye, Xiaoqin Zhou

**Affiliations:** ^1^School of Mental Health and Psychological Sciences, Anhui Medical University, Hefei, China; ^2^Department of Psychiatry, Chaohu Hospital of Anhui Medical University, Hefei, China; ^3^Anhui Psychiatric Center, Hefei, China; ^4^Bengbu Mental Health Center, Anhui Veterans Hospital, Anmin Hospital Affiliated to Bengbu Medical College, Bengbu, China; ^5^Department of Psychology and Sleep Medicine, The Second Affiliated Hospital of Anhui Medical University, Hefei, China

**Keywords:** depression, adolescent, non-suicidal self-injury, childhood trauma, body investment, alexithymia

## Abstract

**Purpose:**

This study analyzes the relationship of alexithymia, childhood trauma, and body investment to non-suicidal self-injury (NSSI) behaviors in adolescents with depressive disorder and whether they have predictive and diagnostic value for non-suicidal self-injury (NSSI) behaviors in adolescents with depressive disorder.

**Patients and methods:**

A total of 225 patients with a diagnosis of adolescent depressive disorder were included in the study and were divided into two groups according to the DSM-5 criteria: 98 cases without NSSI and 127 cases with NSSI. Compare the demographic data, 24-item Hamilton Depression Scale (HAMD-24), 20-item Toronto Alexithymia Scale (TAS-20), Childhood Trauma Questionnaire-Short Form (CTQ-SF), and Body Investment Scale (BIS) scores between two groups. Binary logistic regression was used to analyze the independent risk factors contributing to NSSI behaviors in adolescents with depression, and establish four predictive models. Based on the models’ predictive probability, the ROC curves were plotted to calculate the value of the predictive diagnostic effect.

**Results:**

The group without NSSI had lower scores than the group with NSSI on HAMD-24 total score, TAS-20 total score, difficulty identifying feelings, difficulty describing feelings, and externally focused thinking, as well as lower scores on CTQ-SF total score, physical neglect, emotional neglect, physical abuse, and emotional abuse. In contrast, the BIS total score, body image feelings and attitudes, body care, and body protection factor scores were higher for the group without NSSI. The BIS body care factor score and the CTQ-SF emotional abuse factor score were significantly linked with adolescents diagnosed with depressive disorder who exhibited NSSI behaviors. These results provide a good diagnostic model for adolescents with depressive disorder.

**Conclusion:**

Low levels of body care and childhood emotional abuse may independently contribute to the implementation of NSSI in adolescents with depressive disorder. Body investment and childhood trauma are valuable in diagnosing and predicting NSSI behaviors and should be considered as potentially important factors in clinical treatment.

## Introduction

Non-suicidal self-injury (NSSI) is deliberate injury to body tissues without suicidal intention, which occurs on five or more days within the past year (Buelens et al., 2020; van der Venne et al., 2023). The main forms include scratching, cutting the skin, pulling hair, and hitting oneself, with the most common form being cutting (Halpin and Duffy, 2020). Non-suicidal self-harm is a significant psychiatric problem among adolescents worldwide. During the DSM-5 revision process, NSSI was included as a disease diagnosis. This called for increased attention and more research into the NSSI among adolescents. A recent Meta-analysis found that the global lifetime prevalence of NSSI in children and adolescents is 22.1% (Lim et al., 2019). However, the lifetime prevalence of NSSI in clinical samples can be as high as 80% (Zetterqvist, 2015; Plener et al., 2016). Previous research indicates that depressive disorder and NSSI frequently co-occur (Liang et al., 2023). A survey conducted in China revealed that as many as 55.1% of NSSI patients were diagnosed with depression (Wang et al., 2021). Likewise, about 34.2% of Chinese young people with major depression had a history of self-injury (Kang et al., 2021). Some studies have reported that adolescents with depressive disorder accompanied by NSSI behaviors have significantly higher rates of suicidal ideation than adolescents without self-injurious behaviors (Hepp et al., 2021; Kim et al., 2022). With the increasing prevalence of NSSI in the adolescent population, it has become one of the leading causes of death in young people. Therefore, early identification of risk factors for NSSI in adolescents with depression can prevent adverse events such as death.

Nock’s (2010) integrative theoretical model proposed several psychological and social risk factors that contribute to the development of NSSI behaviors. These factors include both intrapersonal and interpersonal relationships. Several risk factors for NSSI have been identified, including alexithymia, childhood trauma, and low levels of body investment (Liu et al., 2018; Cipriano et al., 2020; Iskric et al., 2020). However, there is still much to be explored about how these factors interact and ultimately lead to the development of NSSI behaviors in adolescents. Alexithymia is a difficulty in regulating emotions, defined as a disorder characterized by a failure to recognize and describe feelings (Dong et al., 2023). Numerous studies have shown a strong association between alexithymia and NSSI (Ying et al., 2023; Zhang et al., 2023). In their study, Tang et al. (2022) discovered that alexithymia has a positive relationship with NSSI and that depression may act as a mediator between alexithymia and NSSI. In addition, Lüdtke et al. (2016) also concluded that alexithymia significantly predicts NSSI in female adolescent hospitalized patients. It is theorized patients who lack the language to express negative emotions (alexithymia) are more likely to express themselves by venting or hurting themselves (self-injury) (Raffagnato et al., 2020). In this respect, alexithymia and NSSI both serve as a pathway for expressing psychological and emotional problems in the adolescents.

Childhood trauma refers to the abusive and traumatizing experiences that an individual suffers before the age of 16. These experiences encompass five forms: physical abuse, emotional abuse, sexual abuse, emotional neglect, and physical neglect (Ying et al., 2023). Childhood trauma may be associated with self-injury in adolescents with depressive disorder. More than one-third of the population has reported experiencing adverse childhood experiences, which is more prevalent among adolescents diagnosed with depression (Zhang et al., 2020). According to Xie et al. (2018), 55.5% of adolescents with depression had suffered at least one form of child abuse. Several studies have suggested that childhood trauma is a risk factor for NSSI in adolescents (Brown and Plener, 2017; Sun et al., 2023). Nock’s (2010) comprehensive theoretical model of self-injury also supports this. According to the theory, many psychological and social factors play a mediating role in childhood trauma and NSSI. For example, in a study of medical students, social support was shown to mediate the relationship between childhood maltreatment and NSSI in only children (Xu et al., 2019). Huang et al. (2022) concluded that psychological sub-health partially mediates the relationship between childhood trauma and NSSI. Additionally, childhood trauma has a positive predictive on self-injury through psychological sub-health. These studies indicate that childhood trauma is another essential influence/predictor of NSSI among adolescents. However, existing reports on the association between different types of childhood trauma and self-injury are inconsistent (Thomassin et al., 2016; Baiden et al., 2017). In this paper, we will evaluate the predictive power of each maltreatment subtype on non-suicidal self-injury after accounting for all available covariates.

Body investment is another important factor that affects the occurrence and development of NSSI, referring to the importance of the body in cognition, behavior and emotion in one’s self-assessment (Marco et al., 2018). This paper employs the Body Investment Scale (BIS) to evaluate the level of body investment in adolescents with depressive disorder. The Body Investment Scale comprises four dimensions: body feelings, body touch, body protection, and body care (Orbach and Mikulincer, 1998). Body investment is associated with self-preservation and represents an attraction to life. This implies active body care and protection. Specifically, individuals may reduce their body investment when experiencing psychological pain or trauma. Such a change influences body’s perception and experience, which may lead to self-harm behaviors (Brausch et al., 2021). Duggan et al. (2013) found that negative attitudes and feelings about the body may be predictors of self-injury and suicide attempts. Research has shown that ninth graders with a history of self-injury and body dissatisfaction are three times more likely to repeat self-injury than those without a history of self-injury (Brunner et al., 2007). Based on this evidence, negative body investment could be a significant factor in self-injury and provides new perspectives for understanding NSSI. To the best of our knowledge, this study is the first cross-sectional research that considers alexithymia, child maltreatment, and body investment as predictors of NSSI in adolescents with depressive disorder.

Therefore, this study aimed to exam the diagnostic and predictive power of alexithymia, childhood trauma, and body investment on NSSI in adolescents with depressive disorder. Based on the available evidence, we propose the following hypotheses: first, alexithymia and childhood trauma are risk factors for NSSI in adolescents with depressive disorder, and body investment is a protective factor for it; Secondly, alexithymia, childhood trauma, and body investment can predict NSSI in adolescents with depressive disorder.

## Materials and methods

### Participants

A total of 225 outpatients or hospitalized adolescents with depressive disorder were included in this study. All participants were recruited through convenience sampling from the Chaohu Hospital of Anhui Medical University (Hefei, Anhui, China) and the Second Affiliated Hospital of Anhui Medical University (Hefei, Anhui, China) between January 2022 and June 2023. Both hospitals are tertiary hospitals directly affiliated with Anhui Medical University, wherein Chaohu Hospital is the psychiatric center of Anhui Province, and both have a large number of adolescents with depressive disorder. Participants must meet the following criteria: (a) 11–19 years old; (b) diagnosed individually by a chief psychiatrist and an attending physician, according to the diagnostic criteria for depressive disorder in the Diagnostic and Statistical Manual of Mental Disorder, fifth edition (DSM-5); (c) the 24-item Hamilton Depression Scale (HAMD-24) score ≥ 8; and (d) able to cooperate in completing relevant questionnaires and scale evaluations for this study. Exclusion criteria: (a) Presence of co-morbidity with other mental disorder, including neurocognitive disorder, substance-related and addiction disorder, schizophrenia, delusional disorder, schizoaffective disorder, transient psychotic disorder, bipolar disorder, intellectual disorder, autism spectrum disorder, eating disorder, borderline personality disorder, hair-pulling fetishism, stereotypical self-injury, and grasping disorder. (b) Presence of co-morbidities with organic brain disorder or other physical diseases; (c) Presence of religious self-injury or customary self-injury. This study was approved by the Ethics Committee of Chaohu Hospital of Anhui Medical University (KYXM-202201-003). All participants and legal guardians gave written consent.

### Procedures

The patient and their legal guardian sign an informed consent form when the patient meets the inclusion criteria and expresses a willingness to participate. Subsequently, the patient was invited to a quiet room for a face-to-face meeting with a psychiatric attending physician and a postgraduate psychiatry student. A self-made general information questionnaire was used to collect data on patients’ age, gender, education level, whether they were the only child, parents’ marital status, family financial situation, history of smoking, and alcohol consumption. NSSI presence or absence is evaluated using the DSM-5 diagnostic criteria for NSSI behaviors. NSSI is defined by fulfilling the criteria: The patient has engaged in three or more non-suicidal self-injury behaviors within the past six months, with at least one occurrence in the last month. The standardized self-report scale was completed autonomously by all participants. Any ambiguities were explained by the interviewer. Then, the questionnaires were coded, and the answers were entered into EpiData before they were exported to a form. Finally, two graduate students in psychiatry reviewed and organized the data.

### Measures

#### The general information questionnaire

A general information questionnaire was self-administered to collect data on participants’ gender, age, level of education, only child, parents’ marital status, father’s level of education, mother’s level of education, family economic status, active smoking, and active drinking.

#### 20-item Toronto Alexithymia Scale

Bagby et al. (1994) developed the scale as a standardized self-report questionnaire for assessing the presence and severity of alexithymia. The scale includes 20 items grouped into 3 dimensions: difficulty identifying feelings, difficulty describing feelings, and externally focused thinking. The rating is based on a 5-point Likert scale, ranging from ‘1 strongly disagree to 5 strongly agree’. The total score of this scale ranges from 20 to 100, with ≤ 50 being no alexithymia, 51–60 being possible alexithymia, and ≥ 61 being the presence of alexithymia, with higher scores representing more severe alexithymia (Franz et al., 2008). The Chinese version of this scale has good reliability and validity indicators (Zhu et al., 2007). In this study, the Cronbach’s α coefficient was 0.83.

#### Childhood Trauma Questionnaire-Short Form

The CTQ-SF is a 28-item self-report questionnaire used to assess individuals’ experiences of childhood abuse and neglect (Bernstein et al., 2003). This questionnaire is composed of five dimensions: emotional abuse, physical abuse, sexual abuse, emotional neglect, and physical neglect. Responses are rated on a 5-point Likert scale ranging from 1 (never) to 5 (always), with higher scores indicating greater severity of abuse and trauma. Each dimension can be categorized as none, mild, moderate or severe based on the score, and a trauma severity of mild and above in any dimension can be defined as positive for trauma in that dimension (Davidson et al., 2009). The Chinese version of the questionnaire has good reliability among Chinese adolescents, with a Cronbach′s α coefficient of 0.73 in this study (He et al., 2019).

#### Body Investment Scale

The BIS is a brief self-report scale that consists of 24 items to assess emotional investment in the body (Orbach and Mikulincer, 1998). The scale contains four subscales: feelings and attitudes toward the body (for example, “I feel anger toward my body”), comfort in physical touch (for example, “I enjoy physical contact with others”), body care (for example, “I pay attention to my appearance”), and body protection (for example, “Sometimes I purposely injure myself”). Each subscale consists of six items. This scale is scored on a 5-point Likert scale from 1 (strongly disagree) to 5 (strongly agree). The scores for the four independent subscales were obtained by averaging all entries for each respective subscale. Scores from all subscales were summed to obtain a total score, with lower scores indicating lower emotional investment in the body. The scale is not available in Chinese, so the English translation was used. Orbach and Mikulincer (1998) validated in adolescents and found good internal consistency for the original scale. In this study, the Cronbach’s α coefficient was 0.78.

#### 24-item Hamilton Depression Scale

The HAMD-24 was developed by Hamilton (1960) in 1960 and is widely used for clinical assessment of depressive states. This study used a 24-item version with seven factors: anxiety/somatization, weight loss, cognitive impairment, day/night changes, retardation, sleep disturbance, and despair. The scale is based on a 5-point system ranging from 0 to 4, with a total of 96 points. A score of less than 8 points indicates a lack of depressive symptoms, while scores between 8–19 points indicate possible depression. Scores between 20–34 points indicate mild or moderate depression, and scores of 35 points or higher indicate severe depression. HAMD-24 has good reliability and validity, and the Cronbach’s α coefficient in this study was 0.90.

### Data analysis

The study’s data underwent statistical analysis using SPSS 26.0. We performed descriptive statistics on the NSSI and non-NSSI groups and calculated the differences between these two groups. Conduct a normality test on the measurement data. If it conforms to the normal distribution, use represents, independent samples *t*-test is used for comparison between groups. Since the TAS-20, CTQ-SF, and BIS all have multiple subscales, we will utilize one-way multivariate ANOVA to compare the scores of these scales between groups. Count data were expressed as frequencies and percentages, while comparisons between groups were performed using the chi-square test. Moreover, binary logistic regression analyses were conducted to examine the risk of independent effects of dysphoria, childhood trauma and physical commitment on NSSI behaviors after adjusting for general demographic information such as gender, age, education, only-child status, parental marital status, parental literacy, and family economic status in order to create predictive models. Based on the predictive probabilities of the models, we plotted the receiver operator characteristic (ROC) curves to evaluate the diagnostic ability of different models to implement the NSSI for adolescents with depressive disorder. Specifically, firstly, the Youden index was computed by subtracting the ROC curve’s Y-axis value from its X-axis value. Subsequently, the maximal Youden index value was found, indicating the optimal critical diagnostic value, also known as the cutoff value, of the ROC curve. Lastly, the corresponding sensitivity and specificity were determined and reported based on the optimal critical value.

## Results

### Comparison of general demographic information and clinical scale scores

A comparison of general demographic data between adolescent depression patients with and without NSSI behaviors is provided in Table 1. Out of the total of cases, there were 98 (43.6%) in the group without NSSI and 127 (56.4%) in the group with NSSI. No statistically significant differences were found between the two groups with regards to only child status, parents’ marital status, father’s education, mother’s education, family economic status, and active smoking (*p* > 0.05). However, significant differences were observed in gender (*χ^2^/t* = 5.13, *p* = 0.024), age (*χ^2^/t* = 2.01, *p* = 0.046), education (*χ^2^/t* = 8.73, *p* = 0.013), and active drinking (*χ^2^/t* = 4.73, *p* = 0.030). Table 2 shows the comparison of the scale scores for the group without NSSI and the group with NSSI. Statistically significant results were obtained for the total and factor scores for the scale, except for body touch in BIS (*F* = 3.86, *p* = 0.051) and sexual abuse in CTQ-SF (*F* = 1.70, *p* = 0.194). Specifically, the group without NSSI showed significant differences in HAMD-24 total score (*t* = −3.89, *p* < 0.001), TAS-20 total score (*F* = 11.92, *p* = 0.001), difficulty identifying feelings (*F* = 6.74, *p* = 0.010), difficulty describing feelings (*F* = 7.53, *p* = 0.007), externally focused thinking (*F* = 4.26, *p* = 0.040), CTQ-SF total score (*F* = 16.97, *p* < 0.001), physical neglect (*F* = 5.73, *p* = 0.017), emotional neglect (*F* = 4.06, *p* = 0.045), physical abuse (*F* = 7.14, *p* = 0.008) and emotional abuse (*F* = 24.21, *p* < 0.001) were lower than the group with NSSI. The BIS total score (*F* = 52.32, *p* < 0.001) and its three factor scores for body feelings (*F* = 26.40, *p* < 0.001), body care (*F* = 78.25, *p* < 0.001), and body protection (*F* = 19.72, *p* < 0.001) were higher than the scores of the group with NSSI.

**Table 1:** Comparison of general demographic data between the group without and with non-suicidal self-injury (NSSI) in adolescents with depressive disorder (*n* = 225).

Variable	Total sample *n* (%)	Without NSSI group *n* (%)	With NSSI group *n* (%)	χ^2^/*t*	*p*
Gender				5.13	0.024*
Boys	63 (28.0)	35 (35.7)	28 (22.0)		
Girls	162 (72.0)	63 (64.3)	99 (78.0)		
Age (years, $\bar{x}\pm s$ )	15.8 ± 1.9	16.0 ± 1.9	15.5 ± 1.9	2.01	0.046*
Educational level				8.73	0.013*
Junior high school and below	93 (41.3)	30 (30.6)	63 (49.6)		
High school or technical secondary school	106 (47.1)	53 (54.1)	53 (41.7)		
College degree or above	26 (11.6)	15 (15.3)	11 (8.7)		
Only child				0.11	0.738
Yes	64 (28.4)	29 (29.6)	35 (27.6)		
No	161 (71.6)	69 (70.4)	92 (72.4)		
Parents’ marital status				0.06	0.807
Married	183 (81.3)	79 (80.6)	104 (81.9)		
Divorce or unilateral survival	42 (18.7)	19 (19.4)	23 (18.1)		
Father’s education level				2.75	0.432
Primary school and below	33 (14.7)	13 (13.3)	20 (15.7)		
Junior high school	106 (47.1)	50 (51.0)	56 (44.1)		
High school or technical secondary school	49 (21.8)	17 (17.3)	32 (25.2)		
College degree or above	37 (16.4)	18 (18.4)	19 (15.0)		
Mother’s education level				1.15	0.766
Primary school and below	58 (25.8)	24 (24.5)	34 (26.8)		
Junior high school	105 (46.7)	44 (44.9)	61 (48.0)		
High school or technical secondary school	37 (16.4)	19 (19.4)	18 (14.2)		
College degree or above	25 (11.1)	11 (11.2)	14 (11.0)		
Family economic status				1.31	0.520
Poor	16 (7.1)	6 (6.1)	10 (7.9)		
Average	195 (86.7)	84 (85.4)	111 (87.4)		
Good	14 (6.2)	8 (8.2)	6 (4.7)		
Active smoking				3.44	0.064
Yes	37 (16.4)	11 (11.2)	26 (20.5)		
No	188 (83.6)	87 (88.8)	101 (79.5)		
Active drinking				4.73	0.030*
Yes	115 (51.1)	42 (42.9)	73 (57.5)		
No	110 (48.9)	56 (57.1)	54 (42.5)		

Differences assessed by Pearson’s chi-square and independent sample *t*-test; *p < 0.05.

**Table 2:** Comparison of scale scores between the group with and without non-suicidal self-injury (NSSI) in adolescents with depressive disorder (*n*=225).

Scale/dimension	without NSSI group (*M*±SD)	with NSSI group (*M*)±SD	*F/t*	*p*
HAMD-24	26.71±9.43	32.02±9.20	−4.24	<0.001***
TAS-20				
Difficulty Identifying feelings	25.73±5.13	27.43±4.61	6.74	0.010*
Difficulty describing feelings	17.71±3.26	18.92±3.28	7.53	0.007**
Externally focused thinking	22.96±3.34	23.95±3.76	4.26	0.040*
Total score	66.41±8.53	70.30±8.27	11.92	0.001**
CTQ-SF				
Physical neglect	9.68±3.24	10.81±3.69	5.73	0.017*
Emotional neglect	15.69±4.65	16.96±4.70	4.06	0.045*
Physical abuse	7.65±3.27	9.06±4.36	7.14	0.008**
Emotional abuse	11.28±4.25	14.19±4.52	24.21	<0.001***
Sexual abuse	5.70±2.24	6.17±2.89	1.70	0.194
Total score	50.01±11.65	57.19±13.88	16.97	<0.001***
BIS				
Body feelings	20.09±4.45	17.18±4.03	26.40	<0.001***
Body touch	14.53±3.87	13.42±4.46	3.86	0.051
Body care	19.63±3.59	15.06±4.03	78.25	<0.001***
Body protection	15.82±3.10	13.83±3.48	19.72	<0.001***
Total score	70.07±10.49	59.49±11.17	52.32	<0.001***

HAMD-24, 24-item Hamilton Depression Scale; TAS-20, 20-item Toronto Alexithymia Scale; CTQ-SF, Childhood trauma questionnaire‑short form; BIS, Body Investment Scale; Differences assessed by independent sample t-test or One-way multivariate analysis of variance; *M*, mean; SD, standard deviation; *p<0.05; **p < 0.01; ***P < 0.001.

### Logistic regression

In multivariate analyses, we used four binary logistic regression models to analyze the relationship between predictor variables and the NSSI among adolescents with depressive disorder. Model 1 included general demographic data as predictor variables for analysis. The model was found to be significant (*χ^2^* = 23.27, *p* < 0.001) with variances of 0.10 (Cox and Snell *R*^2^) and 0.13 (Nagelkerke *R*^2^). Among the variables, gender, level of education, and active drinking significantly predicted NSSI behaviors (*p* < 0.05). Model 2 included three factors from the TAS-20 (difficulty identifying feelings, difficulty describing feelings, and externally focused thinking) and 24-HAMD scores in addition to the variables in Model 1. The model was significant (*χ^2^* = 35.60, *p* < 0.001), and the variance increased to 0.15 (Cox and Snell *R*^2^) and 0.20 (Nagelkerke *R*^2^). Among the variables, education level, active drinking, and 24-HAMD significantly predicted NSSI behaviors (*p* < 0.05). Model 3 included five factors from the CTQ-SF (physical neglect, emotional neglect, physical abuse, emotional abuse and sexual abuse) in addition to the variables in Model 2. The model was significant (*χ^2^* = 47.05, *p* < 0.001), and the variance increased to 0.19 (Cox and Snell *R*^2^) and 0.25 (Nagelkerke *R*^2^). Among the variables, education level, active drinking, 24-HAMD and emotional abuse significantly predicted NSSI behaviors (*p* < 0.05). Model 4 included four factors from the BIS (body feelings, body touch, body care, and body protection) in addition to the variables in Model 3. The final logistic regression model was significant (*χ^2^* = 70.85, *p* < 0.001), and the variance increased to 0.27 (Cox and Snell *R*^2^) and 0.36 (Nagelkerke *R*^2^). Among these variables, emotional abuse (OR = 1.08, 95% CI = 1.00–1.16, *p* = 0.044) and body care (OR = 0.76, 95% CI = 0.67–0.83, *p* < 0.001) significantly predicted NSSI behavior in adolescents with depressive disorders (Table 3).

**Table 3:** Logistic regression predicting non-suicidal self-injury (NSSI) behaviors in adolescents with depressive disorder (*n*=225).

Predictor variables	OR	95% CI	*p*
**Model 1**			
Gender			
Girls	Ref		
Boys	1.95	(1.04,3.67)	0.038*
Educational level			
College degree or above	Ref		
High school or technical secondary school	0.22	(0.08,0.57)	0.002**
Junior high school and below	0.43	(0.23,0.80)	0.008**
Active drinking			
No	Ref		
Yes	2.64	(1.45,4.80)	0.001**
**Model 2**			
Educational level			
College degree or above	Ref		
High school or technical secondary school	0.20	(0.08,0.55)	0.002**
Junior high school and below	0.34	(0.18,0.66)	0.001**
Active drinking			
No	Ref		
Yes	2.27	(1.23,4.21)	0.009**
HAMD-24	1.07	(1.03,1.10)	<0.001***
**Model 3**			
Educational level			
College degree or above	Ref		
High school or technical secondary school	0.19	(0.07,0.53)	0.002**
Junior high school and below	0.37	(0.19,0.71)	0.003**
Active drinking			
No	Ref		
Yes	2.19	(1.16,4.15)	0.016*
HAMD-24	1.05	(1.01,1.08)	0.006**
Emotional abuse	1.13	(1.05,1.21)	0.001**
**Model 4**			
Emotional abuse	1.08	(1.00,1.16)	0.044*
Body care	0.76	(0.67,0.83)	<0.001***

OR, odds ratio; CI, confidence interval; HAMD-24, 24-item Hamilton Depression Scale; all models use stepwise logistic regression, method “Wald forward”; Model 1, general demographic data were included for analysis: gender, age, education level, only child status, father’s education level, mother’s education level, family economic status, active smoking and active drinking; Model 2, in addition to the predictor variables in Model 1, 24-HAMD, difficulty Identifying feelings, difficulty describing feelings, and externally focused thinking were included in the analyses; Model 3, in addition to the predictor variables in Model 2, physical neglect, emotional neglect, physical abuse, emotional abuse and sexual abuse were included in the analyses; Model 4, in addition to the predictor variables in Model 3,body feelings, body touch, body care, body protection were included in the analyses; *p<0.05; **p < 0.01; ***P < 0.001.

### ROC curves for NSSI risk factors prediction models

**Figure 1:**
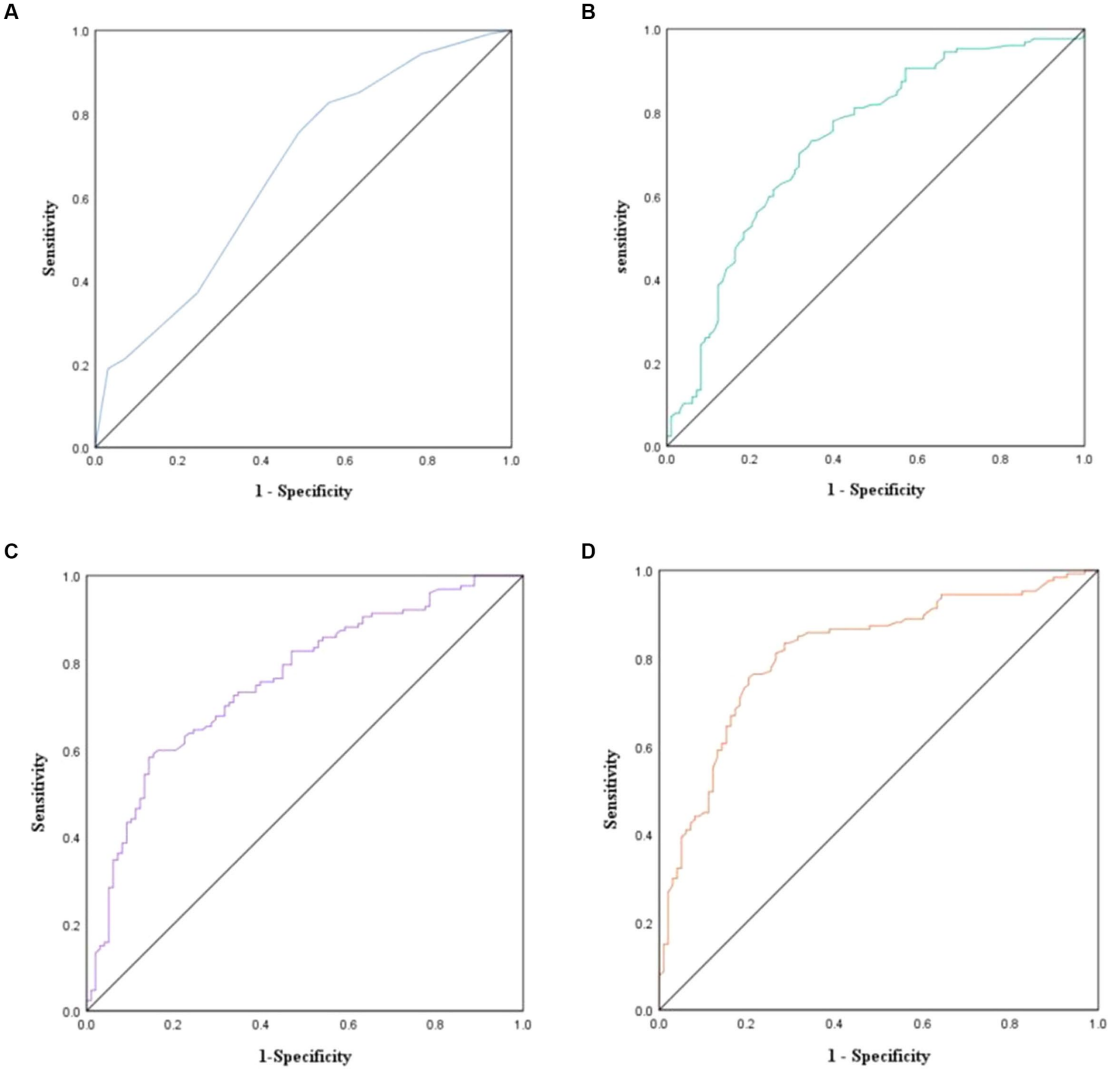
Predictive models for adolescents with depressive disorders with and without non-suicidal self-injurious (NSSI) behaviors ROC curves (*n* = 225). **(A)** ROC curve analysis based on model 1; **(B)** ROC curve analysis based on model 2; **(C)** ROC curve analysis based on model 3; **(D)** ROC curve analysis based on model 4. ROC, Receiver Operating Characteristic.

Four ROC curves were plotted based on the predictive models described above. The area under the ROC curve plotted based on model 1 was 0.66 (SE = 0.04, 95%CI = 0.59–0.73, *p* < 0.001),with an optimal critical point of 0.50, corresponding to sensitivity = 75.6% and specificity = 51.0% (Figure 1A). The area under the ROC curve plotted based on model 2 was 0.73 (SE = 0.03, 95%CI = 0.67–0.80, *p* < 0.001),with an optimal critical point of 0.54, corresponding to sensitivity = 73.2% and specificity = 65.3%(Figure 1B). The area under the ROC curve plotted based on model 3 was 0.76 (SE = 0.03, 95%CI = 0.70–0.82, *p* < 0.001),with an optimal critical point of 0.66, corresponding to sensitivity = 58.3% and specificity = 85.7% (Figure 1C). The area under the ROC curve plotted based on model 4 was 0.82 (SE = 0.03, 95%CI = 0.76–0.87, *p* < 0.001),with an optimal critical point of 0.57, corresponding to sensitivity = 75.6% and specificity = 79.6%(Figure 1D).

## Discussion

This study explored the predictive effects of alexithymia, childhood trauma, and body investment on NSSI behaviors in adolescents with depression and evaluated the diagnostic value of these factors for the occurrence of NSSI in adolescents with depression. Adolescents with depressive disorder and NSSI scored higher than those with depressive disorder without NSSI on HAMD-24, TAS-20 total score, and all its dimensions, CTQ-SF total score, and the other four dimensions except sexual abuse. By contrast, all subscale scores on the BIS, except for body touch, were lower in the group with NSSI compared to the group without NSSI. Our findings support the first hypothesis and are consistent with previous research indicating that childhood trauma and alexithymia increase the risk of NSSI (Kautz et al., 2020; Zhao et al., 2023), while bodily investment serves as a protective factor against NSSI (Pérez et al., 2018). In the final prediction model, childhood trauma and body investment were significant predictors of NSSI behavior, which consistent with previous research (Cipriano et al., 2020; Kautz et al., 2020). Unexpectedly, none of the three narcissistic factors were predictors of NSSI. These findings partially support the second hypothesis.

### General demographic differences between the two groups with and without NSSI’, in order to use more precise and objective language

This study analyzed the general demographic characteristics of depressed adolescents with and without non-suicidal self-injury (NSSI) behaviors. The results indicate that the percentage of girls in the group with NSSI was significantly higher than in the group without NSSI, while the age of adolescents in the former group was significantly lower. Correspondingly, there was a significant difference in the level of education between the two groups. This gender difference supports the results of a meta-analysis that identifies female gender as a risk factor for NSSI (Rahman et al., 2021). However, it has also been demonstrated that gender does not have a significant effect on self-injury (Swannell et al., 2014). The difference in conclusions may be attributed to variations in the definition and assessment of self-injury across different studies (Bresin and Schoenleber, 2015). Males are more likely than females to use more violent methods of self-injury, such as hitting and burning (Sornberger et al., 2012). It is important to note that these methods used by males are often not included in the definition of self-injury. Additionally, due to the smaller sample size of males, precise calculations of male self-harm rates can be challenging. A systematic review of the effects of age on self-injury reported a gradual increase in self-injurious behaviors between 12.5 and 15 years of age, followed by a peak and then a decline (Plener et al., 2015). Our findings regarding the age differences between the two groups of adolescents support this perspective. However, it remains difficult to determine how self-injury in adolescents changes with age. Future research should conduct additional longitudinal studies to explore changing patterns of self-injury at all ages from early adolescence to early adulthood. This finding was consistent with previous research. The group with NSSI exhibited significantly higher levels of active drinking compared to the group without NSSI. This finding supports the conclusion that high alcohol abuse is a significant risk factor for repetitive self-injury (Martiniuk et al., 2015). The reason for this may be that adolescents with self-injurious behaviors are more likely to use alcohol to relieve stress.

### Alexithymia as a risk factor for non-suicidal self-injury in adolescents with depressive disorder

The study found that adolescents in the group with NSSI had significantly higher scores on the TAS-20 total and subscales than those in the group without NSSI. This indicates that alexithymia may be a risk factor for NSSI behaviors in adolescents with depressive disorders. In a longitudinal study of community-based adolescents, it was found that alexithymia scores at baseline were significantly and positively associated with self-injury. Additionally, these scores predicted self-injurious behaviors 5 months later (Garisch and Wilson, 2015). The connection between alexithymia and non-suicidal self-injury can be explained by the emotion regulation model of self-injury. This model suggests that self-injury is a coping mechanism used by individuals to alleviate or reduce negative emotions and free themselves from unwanted emotional states (Klonsky, 2007). Based on this, we should consider alexithymia as an important target for the treatment of non-suicidal self-injurious behaviors in adolescents. Research indicates that mindfulness-based interventions may be an effective approach to alleviate alexithymia (Norman et al., 2019). However, individuals with alexithymia often exhibit primitive defenses and poor empathy, which can make it challenging to achieve positive outcomes with traditional psychotherapy (Casagrande et al., 2020).

### Childhood trauma is associated with increased non-suicidal self-injury in adolescents with depressive disorder

Childhood trauma has been associated with both the lifetime prevalence of NSSI and the recent rise in self-injurious behaviors in community samples of adolescents and clinical samples in adults (Weierich and Nock, 2008; Swannell et al., 2012; Thomassin et al., 2016). Childhood trauma is an independent remote risk factor for NSSI that may cause internal personal vulnerabilities (e.g., emotional regulation difficulties) and interpersonal vulnerabilities (e.g., poor communication skills), thereby increasing the risk of engaging in NSSI (Hooley and Franklin, 2018). More specifically, childhood trauma can lead to an increase in negative emotional responses and even difficulty controlling such emotional responses. To cope with this response, adolescents often resort to using the inappropriate method of NSSI. This is well-supported by the four-function model of self-injury, the biosocial theory of mood disorder, and the integrative theoretical model of the development and maintenance of NSSI (Nock, 2010; Courtney-Seidler et al., 2014). Childhood maltreatment is associated with subsequent neurobiological abnormalities. As adolescents are in a sensitive period of development, childhood trauma can negatively impact them, affecting structural brain development (Kim et al., 2020),diminished HPA axis responses, and circulating inflammatory factors (Klaassens et al., 2009). Due to these neurobiological vulnerabilities, adolescents who have experienced childhood trauma are more likely to develop inadequate coping mechanisms, including NSSI (Westlund Schreiner et al., 2020).

### Body investment as a protective factor for non-suicidal self-injury in adolescents with depressive disorder

Orbach et al. (1995) suggested that a person’s attitude toward protecting their body and investment to their body are key factors in understanding NSSI. He believed that indifference and dissatisfaction with the body leads to a lack of bodily pleasure, which manifests itself in a reduced sensitivity to physical pain. Therefore, when individuals are suffering internally, they are more likely to engage in NSSI and cause harm to their bodies. Hooley et al. (2010) found in a related study that community-based adolescents with a history of self-injury had significantly higher pain tolerance. The longer an individual tolerates pain, the more intense their self-criticism becomes, thereby creating a negative body image. According to clinical experience, Walsh (2012) argues that a person’s relationship and attitude toward their body play a central role in the initiation and maintenance of NSSI. Ross et al. (2009) presented qualitative evidence that adolescents who engaged in NSSI reported more discomfort and dissatisfaction with their bodies. These individuals perceived that their bodies were out of control when compared to adolescents who did not engage in NSSI. It is widely acknowledged that NSSI often takes place in the early to middle stages of adolescence (He et al., 2023). Adolescence is the period when the body is believed to become more significant in one’s self-concept (Markey, 2010),which helps to understand why NSSI manifests itself primarily during this developmental stage. Thus, low levels of bodily investment may be an important risk factor for NSSI and support the inclusion of body investment in etiological models of NSSI.

### Alexithymia does not predictor of non-suicidal self-injury in adolescents with depressive disorder

The logistic regression analyses indicated that none of the factors of alexithymia were significant predictors of NSSI. Adolescents suffering from alexithymia are unable to express their negative emotions verbally and rely on NSSI behaviors to hurt themselves in coping with their psychological pain (Cerutti et al., 2018). This is an unanticipated discovery given the connection between alexithymia and NSSI, as demonstrated in earlier research (Lee, 2016). One reason for this may be the high prevalence of alexithymia in our sample, both in the group with and without NSSI. The difference is likely because we chose young people with depressive disorder as our research participants. Patients with depressive disorder tend to have higher TAS-20 scores and show higher levels of alexithymia (Bordalo and Carvalho, 2022). Furthermore, a study has reported that the relationship between alexithymia and NSSI is fully mediated by depression (Garisch and Wilson, 2010). Consequently, in regression models predicting NSSI behaviors, when both alexithymia and depression are included, alexithymia is no longer a significant predictor. It is possible that depression has a more significant role in NSSI than alexithymia based on this. Although, we cannot ignore the role of alexithymia in non-suicidal self-injury in adolescents with depressive disorders. This is because individuals with depression who also have alexithymia are more likely to engage in self-injury as a way of expressing their feelings or getting rid of unpleasant emotional experiences (Iskric et al., 2020).

### Emotional abuse and physical caregiving as predictors of non-suicidal self-injury in adolescents with depressive disorder

In line with our hypothesis, childhood maltreatment was a notable predictor of NSSI behaviors, which corresponds with the existing literatures (Bunting et al., 2023; Kyei, 2023). Interestingly, there was no significant variation in scores for sexual abuse between the two groups. However, adolescents in the group with NSSI scored slightly higher than those in the group without NSSI. This may be due to the fact that under the influence of traditional Chinese culture, participants were unwilling to reveal excessive information about sexual abuse when accomplishing the scale. In addition, parental education related to sexual bullying and societal awareness of this problem have considerably risen in recent years, leading to a positive outcome in reducing sexual violence among teenagers. After controlling for all available covariates, emotional abuse continued to be an independent risk factor for NSSI in the CTQ-SF. This finding is in keeping with a previous study (Zhang et al., 2022). Similar to our findings, Kang et al. (2018) discovered that emotional abuse was a significant predictor of self-injury in a sample of Chinese adolescents. In contrast, physical abuse was not found to have a substantial impact. Emotional abuse, also called psychological abuse, is a type of emotional harm, unlike physical abuse. It comprises a recurrent behavioral pattern which involves criticism, threats, humiliation, blame, insults, and so on, directed toward children. According to Nock’s (2009) integrative model, experiences of emotional abuse can interfere with a child’s capacity to control intense negative emotions like anger, sadness, fear or shame. Subsequently, the child may adopt NSSI behaviors to assuage these emotions. Previous studies indicated that there is a positive association between childhood emotional abuse and adolescent self-injury (Marco et al., 2018; Zhang et al., 2020). A meta-analysis found that people with a history of childhood emotional abuse had a 3.03 times higher risk for NSSI compared to people with no emotional abuse (Liu et al., 2018). Despite this significance, emotional abuse has garnered less attention in prior research and clinical practice on NSSI than sexual and physical abuse. There is evidence that the pathogenic effects of emotional abuse and other subtypes of abuse on mental health outcomes are similar (Etain et al., 2010; Liu, 2017). As emotional abuse is the most common form of abuse, neglecting emotional abuse may lead to more serious consequences (Stoltenborgh et al., 2015). It is evident that additional attention should be given to emotional abuse within risk assessment and research on NSSI.

Research indicates that negative experiences and perceptions of the body can lead to bodily devaluation and bodily detachment (Muehlenkamp et al., 2013). Aoki et al. (2023) found that trauma can cause individuals to dissociate from their bodies and that NSSI behaviors can be effectively discontinued through the acquisition of self-understanding and stress coping skills. These studies support the role of body attitudes and experiences in the development of non-suicidal self-injury. After controlling for all available covariates, body care remained the most significant predictor of NSSI. Body care refers to taking care of one’s body through enjoyable behaviors such as bathing or using body care products that indicate how much care and attention one pays to one’s body (Brausch and Muehlenkamp, 2007). Body care not only predicts NSSI directly, but it also acts as a mediator for NSSI behaviors. A model of body investment mediating self-injury suggests that all four aspects of body investment mediate the relationship between negative emotions and NSSI in adolescents. This means that adolescents who have negative perceptions of their bodies or neglect them may have a higher likelihood of engaging in NSSI when faced with an overwhelming, aversive emotional state (Muehlenkamp and Brausch, 2012). Body care may be a significant vulnerability factor to consider when attempting to comprehend the risk of NSSI. However, there are also studies that come to the opposite conclusion. A study by Marco et al. (2018) on body investment in patients with eating disorder discovered that body care did not significantly differentiate between patients with self-injury or suicidal ideation. Instead, the remaining three aspects of body investment were independent factors influencing self-injury. Reasons for this discrepancy may include two aspects. Firstly, patients diagnosed with eating disorder predominantly exhibit body checking and body avoidance behaviors in their body care, which are not included in the body care subscale. Secondly, it is crucial to note that our research was conducted on adolescents with depression, and the two research populations are distinct. Yet, there are fewer previous studies related to body care in body investment, and the mechanisms by which body care affects the performance of NSSI in adolescents with depressive disorder need further investigation. In conclusion, we found that low levels of body care were significant predictors of NSSI in adolescents with depressive disorder. Therefore, reconnecting an individual to their body could prove a vital approach in reducing NSSI behaviors. There are studies suggesting that Positive Mindfulness Training’s therapeutic or body acceptance methods have a useful effect on NSSI (Mehlum et al., 2014; Dibaj et al., 2023). In both a 1-year and 3-year follow-up of adolescents exhibiting self-injurious behaviors, Mehlum et al. discovered that those treated with Dialectical Behavior Therapy to Adolescents (DBT-A) showed a substantial and persistent reduction in the frequency of self-injurious behaviors over the long term compared to those receiving Enhanced Usual Care (EUC) (Mehlum et al., 2016, 2019). This indicates that DBT-A could be a viable substitute treatment choice for teenagers experiencing repetitive NSSI behaviors. We ought to assist adolescent patients with low-level investment in the body and concomitant NSSI behaviors to carry out this training, thereby significantly reducing the feelings of body transcendence or objectification required to carry out NSSI.

We applied ROC curves to evaluate the diagnostic efficacy of alexithymia, childhood trauma, and body investment in predicting NSSI behaviors in adolescents with depressive disorder. The results indicate a continuous increase in the area under the ROC curves from Model 1 to Model 4. The sensitivity and specificity of the ROC curves drawn from Models 1 and 2 were smaller than this of the ROC curve drawn from Model 4. Although the ROC curve based on Model 3 had the highest sensitivity among all ROC curves, its specificity was lower. In conclusion, among all ROC curves, the ROC curve based on model 4 was significantly better than the other three curves. This suggests that childhood trauma and body investment have a better predictive diagnostic value for NSSI behaviors in adolescents with depressive disorder. This study’s results have clinical relevance, providing a theoretical foundation for professionals to diagnose NSSI behaviors among depressed adolescents. However, this study primarily focuses on the diagnostic utility of NSSI in adolescent depression patients. The applicability of this model to other NSSI co-morbid patient populations, such as social phobia, post-traumatic stress disorder, borderline personality disorder, and eating disorder, requires further validation through additional research.

### Limitations and future directions

There are some limitations in this study. Firstly, the present study was cross-sectional and could not establish a causal relationship between childhood trauma, body investment and the occurrence of NSSI behaviors in depressed adolescents. Secondly, a healthy control group was not established due to difficulties in finding suitable adolescents who had never engaged in self-injury. This was primarily because the sample collection was conducted among outpatient or inpatient patients. Thirdly, we assessed the NSSI using clinical judgment instead of a structured interview, which may introduce diagnostic bias. Also, we have not included other potential risk factors for self-injury, such as borderline personality traits, mood disorder or other negative emotional states such as anxiety and anger. So, we cannot rule out the possibility that these factors influenced the results of the study. Finally, the data for this survey was collected via questionnaire completion, making recall bias inevitable. Additionally, sensitive questions were included, potentially influencing the accuracy of the information provided by participants. Future research should include more extensive longitudinal studies with independent samples from diverse populations to validate the associations between emotional indifference, childhood trauma, body investment, and NSSI. Additionally, potential mediating or moderating effects among the variables should be further analyzed.

## Conclusion

In summary, the study findings indicate that there is an association between NSSI behaviors in adolescent depression and alexithymia, childhood trauma, and body investment. In logistic regression and ROC analyses, emotional abuse from childhood trauma and body care in terms of body investment were significant predictors of NSSI in this population, with good diagnostic value, allowing us to gain a deeper understanding of the onset and progression of the disease. Therefore, when treating adolescent with NSSI, it is important to consider the role of childhood trauma and levels of body investment in NSSI behaviors. In particular, the significance of body investment should be acknowledged and appreciated as a potential important clinical treatment for adolescent NSSI behaviors. This will enable early intervention to decrease or prevent the perpetration of NSSI behaviors in adolescents with depressive disorder.

## Data availability statement

The raw data supporting the conclusions of this article will be made available by the authors, without undue reservation.

## Ethics statement

Written informed consent was obtained from the individual(s), and minor(s)’ legal guardian/next of kin, for the publication of any potentially identifiable images or data included in this article.

## Author contributions

PC: Conceptualization, Formal analysis, Investigation, Writing – original draft, Writing – review & editing. RP: Conceptualization, Formal analysis, Investigation, Writing – original draft, Writing – review & editing. QY: Conceptualization, Formal analysis, Investigation, Writing – original draft, Writing – review & editing. RZ: Conceptualization, Investigation, Writing – review & editing. MY: Conceptualization, Investigation, Writing – review & editing. XZ: Conceptualization, Investigation, Supervision, Writing – review & editing.
